# Effectiveness of Albumin-Fused Thioredoxin against 6-Hydroxydopamine-Induced Neurotoxicity In Vitro

**DOI:** 10.3390/ijms24119758

**Published:** 2023-06-05

**Authors:** Okina Sakakibara, Mikako Shimoda, Gaku Yamamoto, Youichirou Higashi, Mayumi Ikeda-Imafuku, Yu Ishima, Masahiro Kawahara, Ken-ichiro Tanaka

**Affiliations:** 1 Laboratory of Bio-Analytical Chemistry, Research Institute of Pharmaceutical Sciences, Faculty of Pharmacy, Musashino University, 1-1-20 Shinmachi, Nishitokyo 202-8585, Japanmakawa@musashino-u.ac.jp (M.K.); 2 Department of Pharmacology, Kochi Medical School, Kochi University, Kohasu, Okoh-cho, Nankoku 783-8505, Japan; higasi@kochi-u.ac.jp; 3 Department of Physical Pharmaceutics, School of Pharmaceutical Sciences, Wakayama Medical University, 25-1 Shichiban-Cho, Wakayama 640-8156, Japan; imayu@wakayama-med.ac.jp; 4 Department of Biopharmaceutics, Kyoto Pharmaceutical University, Misasagi, Yamashina-ku, Kyoto 607-8414, Japan; ishimayu@mb.kyoto-phu.ac.jp

**Keywords:** 6-hydroxydopamine, Parkinson’s disease, reactive oxygen species, thioredoxin

## Abstract

Parkinson’s disease (PD) is a neurodegenerative disorder caused by oxidative stress-dependent loss of dopaminergic neurons in the substantia nigra and elevated microglial inflammatory responses. Recent studies show that cell loss also occurs in the hypothalamus in PD. However, effective treatments for the disorder are lacking. Thioredoxin is the major protein disulfide reductase in vivo. We previously synthesized an albumin–thioredoxin fusion protein (Alb–Trx), which has a longer plasma half-life than thioredoxin, and reported its effectiveness in the treatment of respiratory and renal diseases. Moreover, we reported that the fusion protein inhibits trace metal-dependent cell death in cerebrovascular dementia. Here, we investigated the effectiveness of Alb–Trx against 6-hydroxydopamine (6-OHDA)-induced neurotoxicity in vitro. Alb–Trx significantly inhibited 6-OHDA-induced neuronal cell death and the integrated stress response. Alb–Trx also markedly inhibited 6-OHDA-induced reactive oxygen species (ROS) production, at a concentration similar to that inhibiting cell death. Exposure to 6-OHDA perturbed the mitogen-activated protein kinase pathway, with increased phosphorylated Jun N-terminal kinase and decreased phosphorylated extracellular signal-regulated kinase levels. Alb–Trx pretreatment ameliorated these changes. Furthermore, Alb–Trx suppressed 6-OHDA-induced neuroinflammatory responses by inhibiting NF-κB activation. These findings suggest that Alb–Trx reduces neuronal cell death and neuroinflammatory responses by ameliorating ROS-mediated disruptions in intracellular signaling pathways. Thus, Alb–Trx may have potential as a novel therapeutic agent for PD.

## 1. Introduction

Parkinson’s disease (PD) is a progressive neurodegenerative disease affecting the central nervous system and the parts of the body controlled by nerves and is characterized by movement disorders such as resting tremor, akinesia, impaired postural reflexes, and muscle rigidity. Slowness of movement and difficulty walking are typical symptoms seen in the early stages, and cognitive dysfunction and sleep disturbances may also occur. As PD progresses, patients gradually become unable to walk on their own and may become wheelchair-bound or bedridden. Most PD patients are elderly, although it also occurs in younger people, especially in those over 60 years of age, at a rate of about 1 in 100 [1,2]. Generally, the dopamine precursor L-dopa, anticholinergics, and amantadine hydrochloride are used in the treatment of PD to compensate for dopamine depletion due to a decrease in dopamine neurons [3]. While these drugs currently in use have been reported to partially ameliorate symptoms in PD patients, few effective treatments exist to ameliorate the underlying causes of PD.

Although the pathogenesis of PD is not clearly understood, it is thought to be caused by the loss of dopamine neurons in the substantia nigra and the promotion of inflammatory responses by microglia at lesion sites [4,5]. Both in vivo and in vitro studies have used models that mimic oxidative stress-mediated dopaminergic neuronal degeneration and heightened microglial inflammation in which mice or neural cells are treated with 6-hydroxydopamine (6-OHDA) or 1-methyl-4-phenyl-1,2,3,6-tetrahydropyridine [6]. In addition, dysfunction of the hypothalamo–pituitary–adrenal axis has been reported to be involved in the pathogenesis of PD [7]. The hypothalamus controls the secretion of various hormones and is essential for maintaining body homeostasis. Plasma nocturnal growth hormone and adrenocorticotropic hormone levels are decreased in PD patients, suggestive of hypothalamic disturbances [8]. Giguere et al. observed neuronal cell loss in the hypothalamus of patients with PD [9]. Furthermore, there is a significant correlation between the loss of hypocretin and melanin-concentrating hormone neurons in the hypothalamus and the clinical stage of PD [10]. Although the link to dopamine neuron death is not clear, these observations led us to postulate that suppressing neuronal cell death and inflammatory responses in the hypothalamus might be important for treating PD.

Thioredoxin is a low-molecular-weight redox protein present in all organisms that plays an important role in a variety of biological processes [11]. Thioredoxin functions as an antioxidant by promoting the reduction and cleavage of disulfide bonds formed by cysteine residues in proteins. Because of its antioxidant properties, numerous studies have investigated the therapeutic potential of thioredoxin in animal and cellular models of various diseases. For example, Tamaki et al. reported that human thioredoxin-1 ameliorates experimental murine colitis, and that this effect is associated with the suppression of macrophage inhibitory factor production [12]. In addition, others have reported that administration of thioredoxin is effective against myocardial ischemia/reperfusion injury caused by oxidative stress, as well as ethanol-induced oxidative stress, inflammatory cytokine production, and apoptosis in the liver of mice [13,14]. Based on these observations, we hypothesized that thioredoxin might protect against neuronal cell death and inflammation in the 6-OHDA model of PD. However, thioredoxin administered to mice has a short half-life in plasma, of approximately 1 h, because it is rapidly removed by glomerular filtration. Thus, to achieve a therapeutic effect, it must be administered by intravenous infusion or repeated intravenous injection [15,16].

Therefore, to solve this problem, we synthesized an albumin–thioredoxin fusion protein (Alb–Trx), which has a plasma half-life 10-fold greater than thioredoxin [17]. Prior to this study, we performed in vivo analyses using Alb–Trx and found that it is effective against various diseases such as ovalbumin-induced asthma, bleomycin-induced lung fibrosis, and air pollutant-induced lung injury, because of its sustained antioxidant action [18,19,20,21]. Furthermore, we recently found that Alb–Trx protects against excessive trace metal-dependent neuronal cell death in cerebrovascular dementia via its antioxidant properties [22]. In the current study, we analyzed the therapeutic effectiveness of Alb–Trx against neuronal cell death and neuroinflammation in the 6-OHDA mouse model of PD.

## 2. Results

### 2.1. Alb–Trx Inhibits 6-OHDA-Induced Neuronal Cell Death

**Figure 1:**
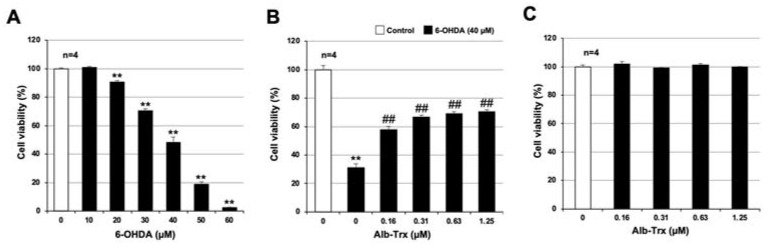
GT1-7 cells were treated with 6-OHDA (10–60 μM) and cultured for a further 24 h (**A**). GT1-7 cells were pretreated with Alb–Trx (0.16–1.25 μM) and then incubated in the absence (Control) or presence of 6-OHDA (40 µM) for 24 h (**B**). GT1-7 cells were treated with Alb–Trx (0.16–1.25 μM) and cultured for a further 24 h (**C**). Viable cell number was determined using CellTiter-Glo^®^ 2.0. Values represent the mean ± S.E.M. (*n* = 4). ** *p* < 0.01, vs. Control; ^##^ *p* < 0.01, vs. 6-OHDA (40 µM) alone.

As described in the introduction, it has been reported that neuronal cell death plays an important role in the onset and exacerbation of PD [4,5]. In addition, PD-like symptoms are induced when 6-OHDA is administered to animals such as mice or rats, and cell death is induced when 6-OHDA is applied to various cell types in vitro [6,23]. First, we analyzed 6-OHDA-induced neuronal cell death in GT1-7 cells, an immortalized mouse hypothalamic neuronal line. To assess viability, intracellular ATP levels were measured using CellTiter-Glo^®^ 2.0. As shown in Figure 1A, treatment with 10–60 μM 6-OHDA reduced cell viability in a concentration-dependent manner. Cell viabilities after treatment with 40, 50 and 60 µM 6-OHDA were 48.3 ± 3.7, 18.9 ± 1.5 and 2.3 ± 0.5% (mean ± S.E.M., *n* = 4), respectively (the viability of the control treated with solvent alone was considered 100%). These results are similar to those reported previously [24]. We next analyzed the effect of Alb–Trx on 6-OHDA-induced neuronal cell death. We used a 6-OHDA concentration of 40 µM, which reduced cell viability to approximately 40%. Alb–Trx was added to the GT1-7 cells immediately before 6-OHDA. Pretreatment with Alb–Trx (0.16–1.25 μM) significantly suppressed the 6-OHDA-dependent decrease in cell viability. Specifically, cell viability in the 6-OHDA + Alb–Trx (1.25 µM) treatment group was about 40% higher than that in the 6-OHDA + PBS treatment group (Figure 1B). Alb–Trx treatment alone did not affect viability (Figure 1C). These results indicate that Alb–Trx inhibits 6-OHDA-induced neuronal cell death in GT1-7 cells.

**Figure 2:**
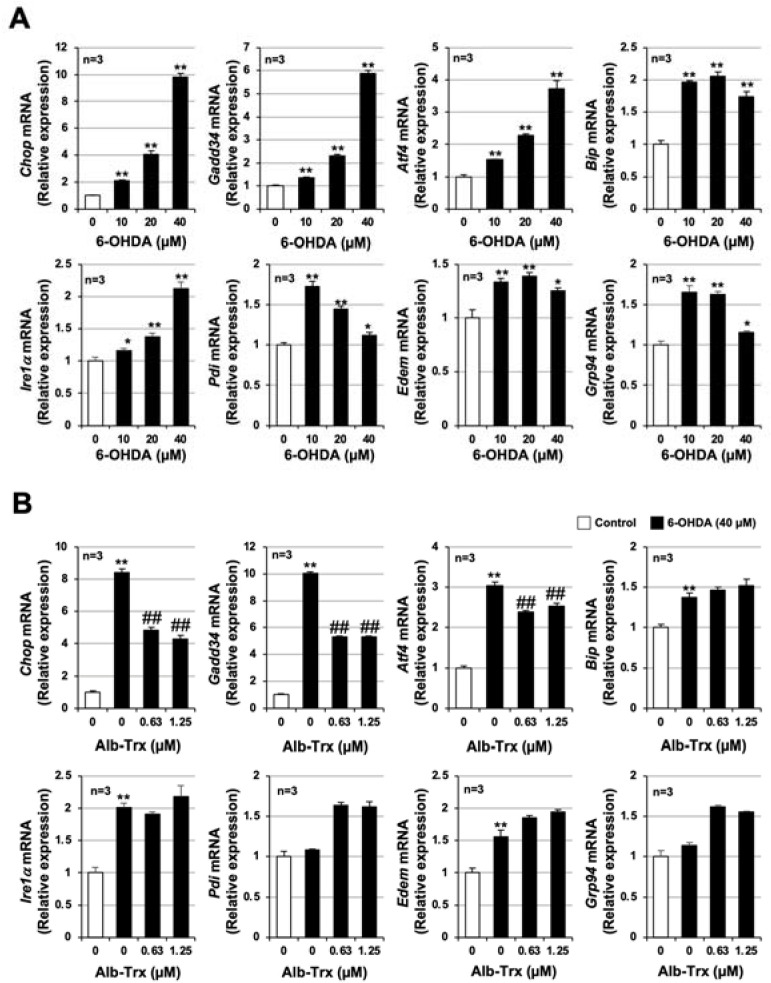
GT1-7 cells were treated with 6-OHDA (10–40 μM) and cultured for 16 h (**A**). GT1-7 cells were pretreated with Alb–Trx (0.63–1.25 μM) and then incubated in the absence (Control) or presence of 6-OHDA (40 µM) for 16 h (**B**). After total RNA extraction from GT1-7 cells, cDNA was synthesized and real-time RT-PCR was performed using primer pairs that specifically amplify *Chop, Gadd34, Atf4, Grp78, Ire1α, Pdi, Edem* and *Grp94*. Values were normalized to *Gapdh* and are expressed relative to the control. Values represent the mean ± S.E.M. (*n* = 3). * *p* < 0.05, vs. Control;** *p* < 0.01, vs. Control; ^##^ *p* < 0.01, vs. 6-OHDA (40 µM) alone.

Previous studies reported that the integrated stress response (ISR) is an important mechanism of 6-OHDA-dependent apoptosis in various cells [25,26]. We recently reported that carnosine, an endogenous dipeptide, inhibits 6-OHDA-induced neuronal cell death by suppressing the expression of ISR-related genes [24]. Thus, we examined the effect of Alb–Trx pretreatment on the 6-OHDA-induced ISR. As shown in Figure 2A, 6-OHDA-induced ISR-related genes, such as CCAAT-enhancer-binding protein homologous protein (*Chop*), growth-arrest and DNA-damage-inducible gene 34 (*Gadd34*), activating transcription factor 4 (*Atf4*), glucose-regulated protein 78 (*Grp78*), inositol-requiring transmembrane kinase/endoribonuclease 1α (*Ire1α*), protein disulfide isomerase (*Pdi*), ER degradation enhancing α mannosidase (*Edem*) and *Grp94*. Among these factors, *Chop*, *Gadd34*, *Atf4* and *Ire1α* were induced in a concentration-dependent manner by 6-OHDA, as was cell death. In comparison, the induction of *Chop*, *Gadd34* and *Atf4* by 6-OHDA was suppressed by Alb–Trx pretreatment (0.63 and 1.25 µM) (Figure 2B). These results suggest that Alb–Trx inhibits 6-OHDA-induced neuronal cell death by suppressing the ISR.

### 2.2. Alb–Trx Inhibits 6-OHDA-Induced Reactive Oxygen Species (ROS) Production

**Figure 3:**
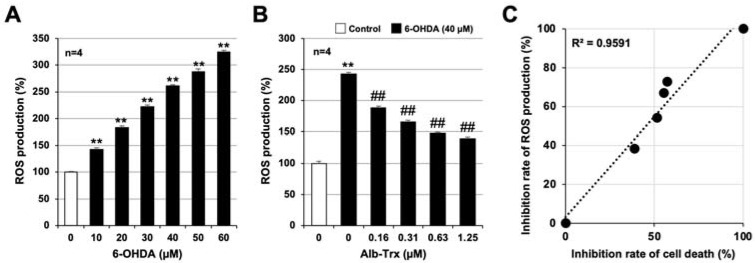
Pre-cultured GT1-7 cells were treated with H_2_DCFDA (10 µM) for 60 min (**A**,**B**). Then, the cells were treated with 6-OHDA (10–60 μM) and cultured for 1 h (**A**). Cells were pre-treated with Alb–Trx (0.16–1.25 μM) and then incubated in the absence (Control) or presence of 6-OHDA (40 µM) for 1 h (**B**). ROS levels were measured using a microplate reader. Correlations between the inhibition of cell death and inhibition of ROS production by Alb–Trx based on the data in Figure 1B and Figure 3B. The inhibition of cell death and ROS production was calculated assuming that treatment with 6-OHDA alone produced 0% inhibition and the control produced 100% inhibition. The coefficient of determination (R^2^) was calculated using Microsoft Excel (version: Microsoft 365) (**C**). Values represent the mean ± S.E.M. (*n* = 4). ** *p* < 0.01, vs. Control; ^##^ *p* < 0.01, vs. 6-OHDA (40 µM) alone.

ROS production is reported to be involved in the onset and progression of PD [27], as well as in 6-OHDA-induced cell death in various cells [28,29]. Indeed, we previously reported that 6-OHDA induces ROS production in GT1-7 cells 2 h after treatment [24]. Therefore, we examined the effect of Alb–Trx on 6-OHDA-induced ROS production in GT1-7 cells using an ROS detection reagent, H_2_DCFDA. 6-OHDA alone promoted ROS production in GT1-7 cells in a concentration-dependent manner (Figure 3A). This effect of 6-OHDA was markedly inhibited by Alb–Trx pretreatment (0.16–1.25 µM) (Figure 3B). Based on the data in Figure 1B and Figure 3B, we show in Figure 3C the correlation between inhibition of cell death and inhibition of ROS production by Alb–Trx. From left to right, data for 6-OHDA alone, 6-OHDA+Alb–Trx (0.16 µM), 6-OHDA+Alb–Trx (0.31 µM), 6-OHDA+Alb–Trx (0.63 µM), 6-OHDA+Alb–Trx (1.25 µM) and Control are plotted in the figure. The mean percent inhibition of 6-OHDA-dependent ROS production in the Alb–Trx pretreatment group was 38.8%, 51.6%, 55.2% and 57.1%, respectively (relative to 100% inhibition for Control and 0% inhibition for 6-OHDA alone) (Figure 3C). These rates of inhibition of ROS production were highly correlated with the rates of inhibition of cell death (Figure 1B) (R^2^ = 0.9591). Furthermore, the inhibitory effect of Alb–Trx on 6-OHDA-dependent ROS production was sustained 24 h after 6-OHDA treatment (Supplementary Figure S1).

**Figure 4:**
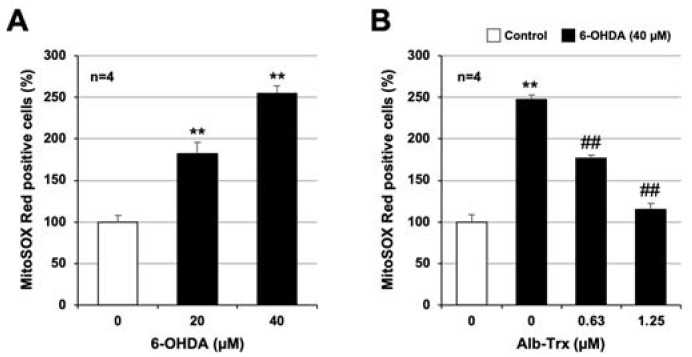
GT1-7 cells were treated with 6-OHDA (10–40 μM) and cultured for 16 h (**A**). GT1-7 cells were pretreated with Alb–Trx (0.63–1.25 μM) and then incubated in the absence (Control) or presence of 6-OHDA (40 µM) for 16 h (**B**). Then, the cells were incubated with a mitochondrial ROS indicator, MitoSOX™ (10 µM), for 30 min, and the mitochondrial ROS levels were measured using a microplate reader. Values represent the mean ± S.E.M. (*n* = 4). ** *p* < 0.01, vs. Control; ^##^ *p* < 0.01, vs. 6-OHDA (40 µM) alone.

We next used MitoSox Red to analyze ROS production in mitochondria. As shown in Figure 4A, 6-OHDA significantly increased ROS production in mitochondria, and this effect was significantly inhibited by Alb–Trx in a dose-dependent manner (Figure 4B). Notably, 6-OHDA-dependent ROS production in mitochondria was almost completely abolished in the Alb–Trx (1.25 µM) pretreatment group. These results strongly suggest that Alb–Trx inhibits 6-OHDA-induced neuronal cell death via suppression of ROS production.

### 2.3. Molecular Mechanisms Linking the Inhibitory Effects of Alb–Trx on ROS Production and Cell Death

Mitogen-activated protein kinases (MAPKs) are serine/threonine kinases involved in a variety of cellular functions including apoptosis, proliferation, division, motility, and inflammatory responses. Three types of MAPKs, c-Jun N-terminal kinase (JNK), extracellular signal-regulated kinase (ERK) and p38, are phosphorylated and activated by specific MAPK kinases (MAPKKs) [30,31]. In addition, several associations between the activation of these MAPKs and oxidative stress have been reported [32,33]. Therefore, we examined the effects of 6-OHDA and Alb–Trx on MAPK activation using antibodies towards the phosphorylated forms of the three different MAPKs.

**Figure 5:**
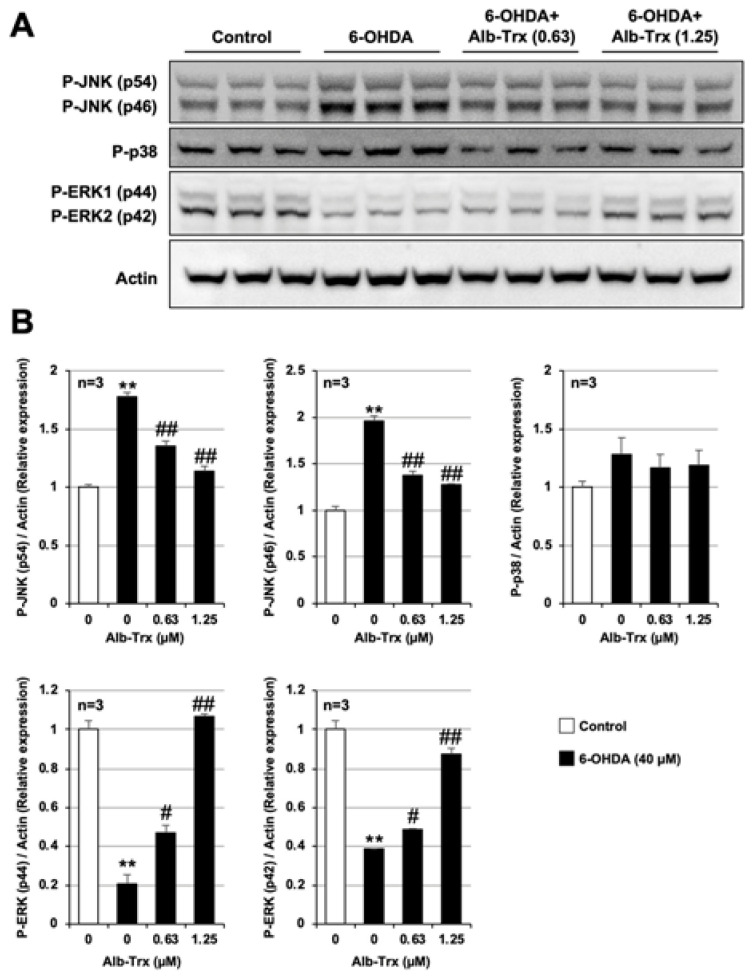
GT1-7 cells were pretreated with Alb–Trx (0.63–1.25 μM) and then incubated in the absence (Control) or presence of 6-OHDA (40 µM) for 2 h. Whole-cell extracts were analyzed by immunoblotting using an antibody against phospho-JNK (P-JNK), phospho-p38 (P-p38), phospho-ERK (P-ERK) or actin (**A**). Band intensities of P-JNK, P-p38 and P-ERK were assessed using ImageJ software (version 1.39u). Values were normalized to the band intensity of actin and are expressed relative to the control (**B**). Values represent the mean ± S.E.M. (*n* = 3). ^#^ *p* < 0.05, vs. 6-OHDA (40 μM) alone; ** *p* < 0.01, vs. Control; ^##^ *p* < 0.01, vs. 6-OHDA (40 µM) alone.

As shown in Figure 5A, the group treated with 6-OHDA alone showed increased phosphorylated JNK (p46 and p54), no change in phosphorylated p38, and decreased phosphorylated ERK (p42 and p44) levels. The increase in phosphorylated JNK and the decrease in phosphorylated ERK were significant by band intensity quantification (Figure 5A). The 6-OHDA-dependent increase in phosphorylated JNK and decrease in phosphorylated ERK were significantly suppressed by Alb–Trx pretreatment. The effect was particularly pronounced in the Alb–Trx (1.25 µM) pretreatment group (Figure 5B). The original images are shown in Supplementary Figure S2. These results suggest that Alb–Trx suppresses 6-OHDA-dependent cell death, at least in part, by countering its effects on JNK and ERK activation.

### 2.4. Alb–Trx Inhibits 6-OHDA-Induced Inflammatory Responses

**Figure 6:**
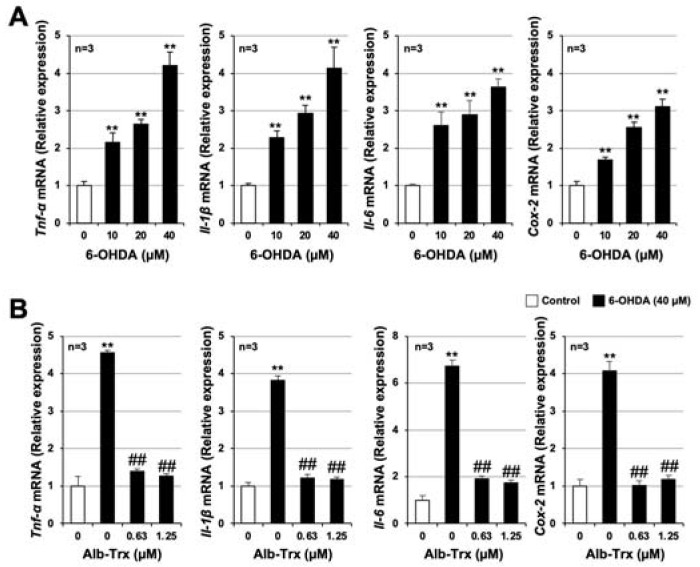
GT1-7 cells were treated with 6-OHDA (10–40 μM) and cultured for 6 h (**A**). GT1-7 cells were pretreated with Alb–Trx (0.63–1.25 μM) and then incubated in the absence (Control) or presence of 6-OHDA (40 µM) for 6 h (**B**). After total RNA extraction from GT1-7 cells, cDNA was synthesized and real-time RT-PCR was performed using primer pairs that specifically amplify *Tnf-α, Il-1β, Il-6 and Cox-2*. Values were normalized to *Gapdh* and are expressed relative to the control. Values represent the mean ± S.E.M. (*n* = 3). ** *p* < 0.01, vs. Control; ^##^ *p* < 0.01, vs. 6-OHDA (40 µM) alone.

Excessive inflammatory responses, as well as increased neuronal cell death, contribute significantly to the development and progression of PD [5]. We previously reported that Alb–Trx exerts anti-inflammatory effects in lung injury models such as pulmonary fibrosis and air pollutant-induced lung injury [18,19,20,21]. Therefore, we examined the effects of Alb–Trx on 6-OHDA-dependent inflammatory responses using GT1-7 cells. As shown in Figure 6A, pro-inflammatory cytokines, such as tumor necrosis factor α (*Tnf-α*), interleukin 1β (*Il-1β*), *Il-6* and cyclooxygenase 2 (*Cox2*), were induced by 6-OHDA treatment. Pretreatment with Alb–Trx (0.63–1.25 µM) almost fully blocked these changes (Figure 6B).

**Figure 7:**
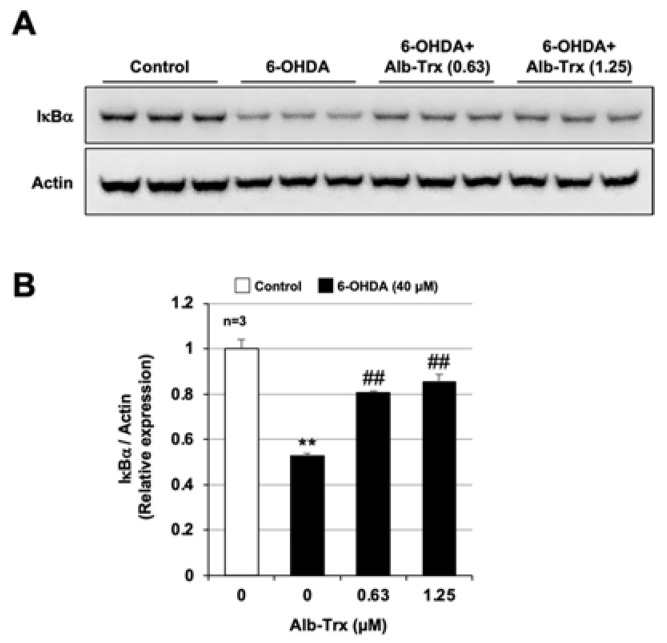
GT1-7 cells were pre-treated with Alb–Trx (0.63–1.25 μM) and then incubated in the absence (Control) or presence of 6-OHDA (40 µM) for 6 h. Whole-cell extracts were analyzed by immunoblotting using an antibody against IκBα or actin (**A**). Band intensities of IκBα were determined using ImageJ software. Values were normalized to the band intensity of actin and are expressed relative to the control (**B**). Values represent the mean ± S.E.M. (*n* = 3). ** *p* < 0.01, vs. Control; ^##^ *p* < 0.01, vs. 6-OHDA (40 µM) alone.

We next analyzed the activation of NF-κB, a key factor in the inflammatory response, by measuring the degradation of IκBα. Whole-cell extracts were collected and analyzed for IκBα protein expression 6 h after 6-OHDA treatment. 6-OHDA reduced IκBα in GT1-7 cells by about half (relative expression = 0.53 ± 0.01) (Figure 7A). Pretreatment with Alb–Trx significantly suppressed this 6-OHDA-mediated downregulation of IκBα (Figure 7B). The original images are shown in Supplementary Figure S3. We further analyzed the effect of Alb–Trx on 6-OHDA-dependent oxidative stress in microglia, a key player in the inflammatory response in PD [34]. As shown in Supplementary Figure S4, in BV2 cells, a microglial cell line, 6-OHDA-dependent ROS production was markedly suppressed by Alb–Trx, similar to GT1-7 cells. These results suggest that Alb–Trx suppresses 6-OHDA-dependent inflammatory responses via inhibition of NF-κB activation.

## 3. Discussion

We conducted this study to determine the effects of Alb–Trx on 6-OHDA-induced neuronal cell death and neuroinflammatory responses using GT1-7 cells, immortalized mouse hypothalamic neurons. Firstly, we showed that Alb–Trx inhibits 6-OHDA-induced cell death and upregulation of ISR-related factors, including *Chop*, *Gadd34* and *Atf4*, in GT1-7 cells. In general, ISR signaling is triggered by phosphorylation of eukaryotic translation initiation factor 2α (eIF2α), which is a common substrate of four different kinases activated by various stress signals, such as viral infection, amino acid deprivation, heme deprivation and ER stress. This, in turn, induces translation of ISR-specific mRNAs, such as ATF4, which then activates transcription of *Chop* and *Gadd34*, which promote cell death induction in various cells [35,36]. In addition, all four kinases (double-stranded RNA-dependent protein kinase, PKR-like endoplasmic reticulum kinase, general control nonderepressible 2 kinase, and heme-regulated eIF2α kinase) that activate eIF2α, the core component of the ISR, are activated by oxidative stress [37,38,39]. Thus, our results suggest that Alb–Trx, with its antioxidant properties, inhibits 6-OHDA-dependent neuronal cell death by suppressing induction of ISR-related factors, especially *Chop*, *Gadd34* and *Atf4*. In contrast, since this study is an in vitro analysis using hypothalamic neurons and microglia, it is not possible to consider cell–cell interactions, the disposition of Alb–Trx in the body, or the effects of interactions between Alb–Trx and other proteins in the body. Therefore, our future studies should use in vivo models to prove the efficacy of Alb–Trx against PD.

6-OHDA upregulated phosphorylated JNK, but Alb–Trx robustly suppressed this increase, to near control levels. Various environmental stresses, including oxidative stress, inflammatory cytokines and metals, activate the JNK signaling pathway [40,41,42]. Apoptosis signal-regulated kinase 1 (ASK1), a member of the MAPK kinase kinase family, activates both MKK4 and MKK7, and these two kinases phosphorylate JNK. Normally, endogenous Trx binds to the N-terminal region of ASK1 and inhibits its activity, but upon oxidative stress or other stimuli, Trx dissociates from ASK1, resulting in its activation by autophosphorylation of its kinase domain [43,44]. Therefore, it is likely that Alb–Trx suppresses 6-OHDA-dependent ASK1 phosphorylation and JNK activation via inhibition of ROS production. In contrast, it has been reported that activation of Rho kinase is required for JNK activation in human lung microvascular endothelial cells [45], and the possibility that other pathways may be involved in JNK activation should be considered. Interestingly, under our experimental conditions, phosphorylated ERK levels were markedly reduced by 6-OHDA, and Alb–Trx countered this change. Typically, activated ERK1/2 phosphorylates RSK, and both RSK and ERK translocate to the nucleus, where they activate multiple transcription factors that promote cell growth and survival [46]. The importance of ERK activity in the regulation of cyclin D1 levels, a protein required for cell cycle progression, and DNA synthesis has been reported using human cultured airway smooth muscle [47]. Moreover, it has been reported that inhibition of phospholipase A2-induced ROS generation markedly restores phosphorylated ERK levels and improves cell viability in U937 cells, suggesting that oxidative stress may lead to ERK inactivation [32]. Additionally, curcin C, extracted from *Jatropha curcas* seeds, has been shown to reduce the expression of phosphorylated ERK via ROS production [48]. Therefore, we speculate that Alb–Trx may counteract ERK inactivation by inhibiting 6-OHDA-dependent ROS production.

Excessive neuroinflammatory responses contribute significantly to the onset and progression of PD [5]. In this study, we found that Alb–Trx inhibits not only 6-OHDA-dependent induction of cell death, but also neuroinflammatory responses. Furthermore, we suggest that this action of Alb–Trx is mediated, at least in part, by the suppression of the degradation of IκBα, which is required for NF-κB activation. IκB kinase, which acts upstream of the NF-κB pathway, is known to be activated by oxidative stress. Whether ROS activates the NF-κB signaling pathway is controversial [49], but we speculate that under our experimental conditions, ROS may promote NF-κB activation. For example, analysis of IKK (kinases involved in the degradation of IκB) mutants has revealed that oxidative stress activates NF-κB through the activation of IKK via phosphorylation of serine residues in the activation loops [50]. In addition, Takada et al. found that H_2_O_2_, an oxidant, induces NF-κB activation by tyrosine phosphorylation of IκBα via spleen tyrosine kinase. Based on these reports, we posit that the inhibitory effect of Alb–Trx on 6-OHDA-induced ROS production may contribute to the inhibition of NF-κB activation, although further analysis is needed.

Oxidative stress is known to be one of the most important factors involved in the development and exacerbation of PD [27,28,29]. Specifically, in one study, endogenous plasma lipid peroxidation, a marker of oxidative stress, was measured by spectrofluorometry in 52 sporadic PD patients and 40 controls, demonstrating that lipid peroxidation levels were significantly (33%) higher in the PD group than in the control group [51]. Another team analyzed 8-hydroxyguanine (8-OHG), a DNA damage product associated with ROS, in controls and PD brains by gas chromatography/mass spectrometry, and found that 8-OHG levels tended to be elevated in PD [52]. Furthermore, analysis of oxidative stress in sera of 40 PD patients and 46 controls showed that oxidative stress markers (thiobarbituric acid-reactive substances and advanced oxidation protein products) were significantly higher in PD patients, while antioxidant markers (ferric reducing ability of plasma, vitamin C, and non-protein thiols) were significantly lower in PD patients [53]. Furthermore, it is interesting to note that in the present study, ROS production in mitochondria was significantly suppressed by Alb–Trx treatment. In fact, mitochondria are the major production site of ROS in cells, and approximately 1–3% of mitochondrial oxygen consumption is converted to ROS [54]. Furthermore, impairment of complex-I activity and high levels of somatic mtDNA point mutations in PD patients have been reported [55,56]. These observations support our contention that Alb–Trx, which can inhibit excessive ROS production, may be a promising treatment for PD.

We fused thioredoxin with albumin not only because albumin is non-toxic, non-immunogenic and less susceptible to glomerular filtration, but also to enable active uptake mediated by gp60, an endothelial cell membrane 60-kDa albumin-binding protein localized in caveolae [57]. However, it is difficult for proteins such as Alb–Trx to cross the blood–brain barrier (BBB), and the expression of gp60 has not been confirmed in the brain [58]. In contrast, cellular uptake of albumin can be enhanced by cationic modification. For example, cationic serum albumin is considered a brain-targeting carrier because the interaction between the positive charge around albumin and the negative surface of the brain capillary endothelium allows it to pass through the BBB [59,60,61]. Given these observations and our current findings, we intend to construct a cationic Alb–Trx fusion protein using site-directed mutagenesis and analyze its efficacy in animal models of PD. Cationic Alb–Trx should more readily cross the BBB, and thus may be useful as a novel therapeutic agent for PD as well as other brain diseases.

## 4. Materials and Methods

### 4.1. Chemicals and Reagents

6-OHDA was purchased from R&D Systems, Inc. (Minneapolis, MN, USA). CellTiter-Glo^®^ 2.0 was purchased from Promega Corporation (Madison, WI, USA). Dulbecco’s Modified Eagle’s Medium/Ham’s Nutrient Mixture F-12 (DMEM/Ham’s-F12) and low-glucose DMEM were purchased from Fujifilm Wako Pure Chemical Corporation (Tokyo, Japan). FastGene™ RNA Basic kit was obtained from Nippon Genetics Co., Ltd. (Tokyo, Japan), PrimeScript^™^ RT master mix (Perfect Real Time) was obtained from Takara Bio (Shiga, Japan), and THUNDERBIRD^®^ Next SYBR^®^ qPCR mix was obtained from Toyobo (Osaka, Japan). 2′,7′-dichlorodihydrofluorescein diacetate (H_2_DCFDA) was obtained from Merck KGaA (Darmstadt, Germany), and MitoSOX™ was obtained from Thermo Fisher Scientific (Waltham, MA). Antibody against actin (Code: SC-47778) was purchased from Santa Cruz Biotechnology (Santa Cruz, CA, USA). Antibodies against phospho-JNK (p46 and p54) (Code: #4668), phospho-p38 (Code: #4511), phospho-ERK (Code: #4376), and IκBα (Code: #4814) and goat anti-rabbit IgG, HRP-linked antibody (Code: #7074) were purchased from Cell Signaling Technology Japan (Tokyo, Japan). HRP-conjugated donkey anti-mouse IgG was purchased from GE Healthcare (Tokyo, Japan).

### 4.2. Preparation of the Alb–Trx Fusion Protein

Mouse serum albumin and the Alb–Trx fusion protein were produced following previously reported methods with slight modification [20,62]. In brief, transformed *Pichia pastoris* cells were preincubated in 100 mL BMGY liquid media (growth phase) for 1 day at 20 °C and then cultured in 10 L of BMMY medium supplemented with 1% casamino acids (protein induction phase) for 7 days at 20 °C. Then, the medium was filtered through a 0.45 μm filter, and the fusion protein was purified by chromatography on a Blue Sepharose 6 Fast Flow column and HiTrap Phenyl HP column by hydrophobic chromatography. The fusion protein was analyzed by SDS-PAGE using a 10% polyacrylamide gel, with Coomassie blue R250 staining. The purity of the fusion protein was estimated to be in excess of 95%.

### 4.3. Cell Culture

GT1-7 cells (immortalized mouse hypothalamic neurons) provided by Dr. R. Weiner (University of California, San Francisco, CA, USA) were cultured in DMEM/Ham’s-F12 supplemented with 10% fetal bovine serum (Biowest, Nuaillé, France). BV2 cells (mouse microglial cells) were purchased from the American Type Culture Collection (Manassas, VA, USA) and cultured in low-glucose DMEM supplemented with 10% fetal bovine serum (heat-inactivated). After trypsin (Fujifilm Wako Pure Chemical Corporation) treatment, the cells were resuspended in serum-free medium, seeded onto culture dishes, and cultured in a humidified incubator (7% or 5% CO_2_) at 37 °C [63].

### 4.4. Measurement of Viable Cell Number

Viable cell number was measured as previously described [64,65]. Briefly, after trypsin treatment, GT1-7 cells were seeded onto 96-well culture plates at a concentration of 3.0 × 10^4^ cells/well in 100 μL culture medium. After pre-incubation for 24 h, cells were treated with Alb–Trx (0.16–1.25 μM) prior to the addition of 6-OHDA (40 μM) to the medium. After 24 h of exposure to the reagents, viable cell number was quantified using CellTiter-Glo^®^ 2.0.

### 4.5. Measurement of ROS Levels

GT1-7 cells or BV2 cells were pre-cultured in black 96-well microplates (3.0 × 10^4^ cells/well or 2.0 × 10^4^ cells/well) for 24 h. Thereafter, the cells were incubated with the ROS indicator H_2_DCFDA (10 µM) for 60 min. The cells were then treated with Alb–Trx (0.16–1.25 μM or 0.16–2.5 μM) prior to the addition of 6-OHDA (40 µM or 60 μM) to the medium. After 1 and 24 h, the ROS levels were measured using a microplate reader (Tecan, Kawasaki, Japan; excitation: 480 nm, emission: 530 nm).

### 4.6. Measurement of Mitochondrial ROS Levels

GT1-7 cells were pre-cultured in black 96-well microplates (3.0 × 10^4^ cells/well) for 24 h, and then treated with Alb–Trx (0.16–1.25 μM) prior to the addition of 6-OHDA (40 µM) to the medium. After 16 h, the cells were incubated with the mitochondrial ROS indicator MitoSOX™ (10 µM) for 30 min. Mitochondrial ROS levels were measured using a microplate reader (Tecan, Kawasaki, Japan; excitation: 510 nm, emission: 580 nm).

### 4.7. Real-Time Reverse-Transcription Polymerase Chain Reaction (RT-PCR) Analysis

Total RNA was extracted from GT1-7 cells using FastGene™ RNA Basic kit in accordance with the manufacturer’s protocol. The samples were reverse-transcribed using the PrimeScript RT master mix, and the resulting cDNA was used in the real-time PCR experiments with THUNDERBIRD Next SYBR qPCR mix, and analyzed with a Bio-Rad (Hercules, CA, USA) CFX96™ real-time system and CFX Manager™ software (Version 3.1). Specificity was confirmed by electrophoretic analysis of the reaction products and the inclusion of template- or reverse-transcriptase-free controls. To normalize the amount of total RNA present in each reaction, glyceraldehyde-3-phosphate dehydrogenase (*Gapdh*) cDNA was used as an internal standard. Primers were designed using the Primer-BLAST website (https://www.ncbi.nlm.nih.gov/tools/primer-blast/, accessed on 1 April 2023). Primer sequences will be provided upon request.

### 4.8. Western Blotting

GT1-7 cells grown in 6-well culture plates (8.1 × 10^5^ cells/well) were lysed with RIPA buffer containing protease and phosphatase inhibitors (Code: 87786 and 78420, Thermo Fisher Scientific). Protein concentration was measured using Bradford Reagent (Takara Bio). Samples were applied to NuPAGE^®^ Novex 4%–12% Bis-Tris Protein Gels (Thermo Fisher Scientific), electrophoresed at a constant voltage of 180 V, and proteins transferred to polyvinylidene difluoride (PVDF) membranes (Code: IB24002, Thermo Fisher Scientific) using the iBlot^®^ 7-Minute Blotting System (Thermo Fisher Scientific). Membranes were blocked with 5% non-fat dry milk at room temperature for 1 h, and incubated with rabbit phospho-JNK antibody (1:1000 dilution), rabbit phospho-p38 antibody (1:1000 dilution), rabbit phospho-ERK antibody (1:1000 dilution), mouse IκBα antibody (1:1000 dilution) or mouse actin antibody (1:1000 dilution) in 5% bovine serum albumin (BSA), 1× Tris-buffered saline (TBS) and 0.1% Tween-20 overnight, followed by incubation with a goat anti-rabbit HRP-linked IgG (1:2000 dilution) or a horse anti-mouse HRP-linked IgG (1:4000 dilution) in 1× TBS and 0.1% Tween-20 for 1 h. Protein bands were visualized using SuperSignal™ West Dura Extended Duration Substrate (Thermo Fisher Scientific). Band intensities were quantitated using ImageJ software (version 1.39u) and normalized to actin.

### 4.9. Statistical Analysis

All data are expressed as the mean ± S.E.M. Significant differences between groups were examined using one-way of analysis of variance (ANOVA) followed by Dunnett’s test. Mac statistical analysis Ver. 3.0 software (Esumi Co., Ltd., Tokyo, Japan) was used for all statistical analyses. Differences were considered to be significant when *p* < 0.05 (* or # *p* < 0.05, ** or ## *p* < 0.01). Details of the symbols are given in the respective figure legends.

## 5. Conclusions

In summary, we found that Alb–Trx inhibits 6-OHDA-induced neuronal cell death and neuroinflammatory responses. Furthermore, Alb–Trx markedly suppressed 6-OHDA-induced ROS production. These findings demonstrate that Alb–Trx suppresses neuronal cell death and neuroinflammatory responses by improving ROS-mediated disruptions in intracellular signaling pathways. Therefore, Alb–Trx may have potential as a novel therapeutic agent for PD.

## Data Availability

The data that support the findings of our study are available from the corresponding author upon reasonable request.

## Supplementary Materials

The supporting information can be downloaded at https://www.mdpi.com/article/10.3390/ijms24119758/s1.
