# Supplementary material for: Effectiveness of Albumin-Fused Thioredoxin against 6-Hydroxydopamine-Induced Neurotoxicity In Vitro
Source: Int J Mol Sci. 2023 Jun 5;24(11):9758. doi: 10.3390/ijms24119758 (PMC10253867; doi:10.3390/ijms24119758)
Supplement: Supplementary file 1 [file ijms-24-09758-s001.zip › ijms-2373394-supplementary.pdf]

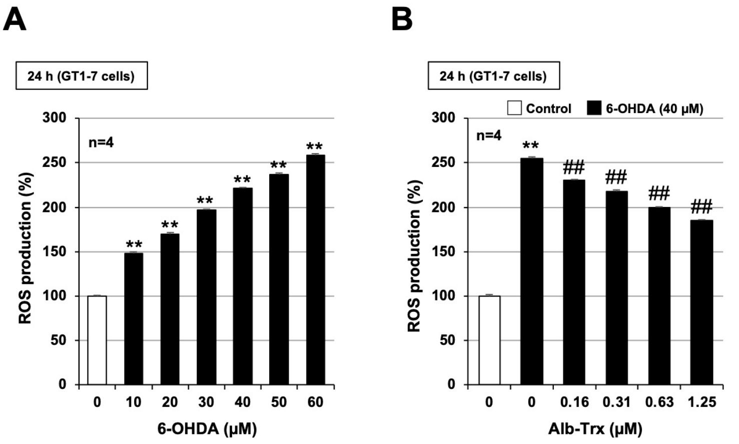

Supplementary Figure S1. Inhibitory effect of Alb-Trx on ROS production 24 h after 6-OHDA treatment. GT1-7 cells were treated with H_2DCFDA (10 μM) for 60 min (**A, B**). Then, the cells were treated with 6-OHDA (10–60 μM) and cultured for 24 h (A). Cells were pre-treated with Alb-Trx (0.16–1.25 μM) and then incubated in the absence (Control) or presence of 6-OHDA (40 μM) for 24 h (B). The ROS levels were measured using a microplate reader. Values represent the mean ± S.E.M. (n = 4). $^{**}P < 0.01$, vs Control; $^{\#\#}P < 0.01$, vs 6-OHDA (40 μM) alone.

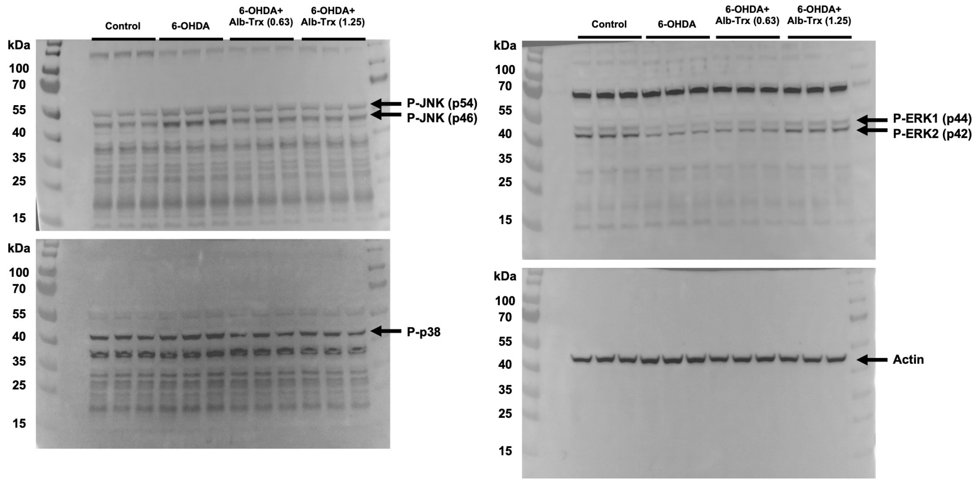

Supplementary Figure S2. Original images for western blotting analysis (Figure 5).

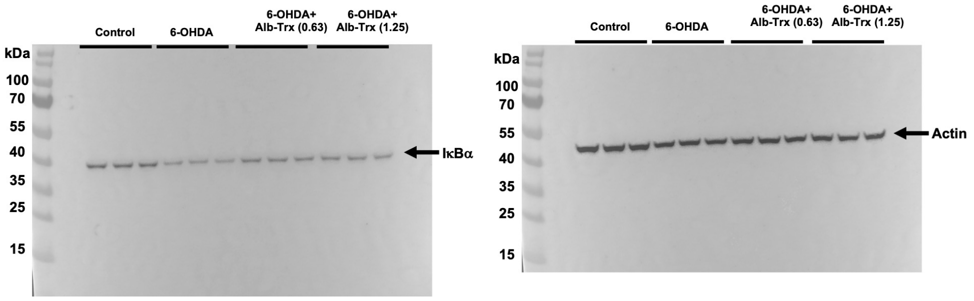

Supplementary Figure S3. Original images for western blotting analysis (Figure 7).

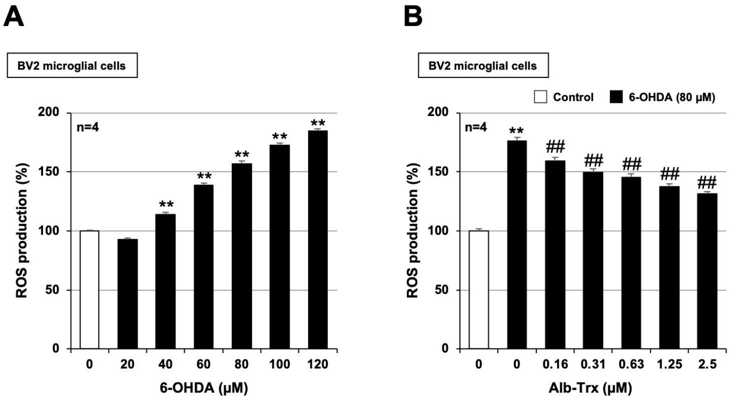

Supplementary Figure S4. Alb-Trx inhibits 6-OHDA-induced ROS production in BV2 microglial cells. BV-2 cells were treated with H_2DCFDA (10 μM) for 60 min (**A, B**). Then, the cells were treated with 6-OHDA (20–120 μM) and cultured for 24 h (A). Cells were pre-treated with Alb-Trx (0.16–2.5 μM) and then incubated in the absence (Control) or presence of 6-OHDA (80 μM) for 24 h (B). The ROS levels were measured using a microplate reader. Values represent the mean ± S.E.M. (n = 4). $^{**}P < 0.01$, vs Control; $^{\#\#}P < 0.01$, vs 6-OHDA (40 μM) alone.
